# Age-Related Decline of Wrist Position Sense and its Relationship to Specific Physical Training

**DOI:** 10.3389/fnhum.2017.00570

**Published:** 2017-11-21

**Authors:** Ann Van de Winckel, Yu-Ting Tseng, Daniel Chantigian, Kaitlyn Lorant, Zinat Zarandi, Jeffrey Buchanan, Thomas A. Zeffiro, Mia Larson, Becky Olson-Kellogg, Jürgen Konczak, Manda L. Keller-Ross

**Affiliations:** ^1^Brain Plasticity Laboratory, Division of Physical Therapy and Division of Rehabilitation Science, Department of Rehabilitation Medicine, Medical School, University of Minnesota, Minneapolis, MN, United States; ^2^Human Sensorimotor Control Laboratory, School of Kinesiology, University of Minnesota, Minneapolis, MN, United States; ^3^Division of Rehabilitation Science, Department of Rehabilitation Medicine, Medical School, University of Minnesota, Minneapolis, MN, United States; ^4^Illinois Bone and Joint Institute, Chicago, IL, United States; ^5^Neurometrika, Potomac, MD, United States; ^6^Division of Physical Therapy, Department of Rehabilitation Medicine, Medical School, University of Minnesota, Minneapolis, MN, United States; ^7^Cardiovascular Research and Rehabilitation Laboratory, Division of Physical Therapy and Division of Rehabilitation Science, Department of Rehabilitation Medicine, Medical School, University of Minnesota, Minneapolis, MN, United States

**Keywords:** proprioception, position sense, wrist, adult, exercise, dancing, human, sensorimotor

## Abstract

Perception of limb and body positions is known as proprioception. Sensory feedback, especially from proprioceptive receptors, is essential for motor control. Aging is associated with a decline in position sense at proximal joints, but there is inconclusive evidence of distal joints being equally affected by aging. In addition, there is initial evidence that physical activity attenuates age-related decline in proprioception. Our objectives were, first, to establish wrist proprioceptive acuity in a large group of seniors and compare their perception to young adults, and second, to determine if specific types of training or regular physical activity are associated with preserved wrist proprioception. We recruited community-dwelling seniors (*n* = 107, mean age, 70 ± 5 years, range, 65–84 years) without cognitive decline (Mini Mental State Examination-brief version ≥13/16) and young adult students (*n* = 51, mean age, 20 ± 1 years, range, 19–26 years). Participants performed contralateral and ipsilateral wrist position sense matching tasks with a bimanual wrist manipulandum to a 15° flexion reference position. Systematic error or *proprioceptive bias* was computed as the mean difference between matched and reference position. The respective standard deviation over five trials constituted a measure of random error or *proprioceptive precision*. Current levels of physical activity and previous sport, musical, or dance training were obtained through a questionnaire. We employed longitudinal mixed effects linear models to calculate the effects of trial number, sex, type of matching task and age on wrist proprioceptive bias and precision. The main results were that relative proprioceptive bias was greater in older when compared to young adults (mean difference: 36% ipsilateral, 88% contralateral, *p* < 0.01). Proprioceptive precision for contralateral but not for ipsilateral matching was smaller in older than in young adults (mean difference: 38% contralateral, *p* < 0.01). Longer years of dance training were associated with smaller bias during ipsilateral matching (*p* < 0.01). Other types of training or physical activity levels did not affect bias or precision. Our findings demonstrate that aging is associated with a decline in proprioceptive bias in distal arm joints, but age does not negatively affect proprioceptive precision. Further, specific types of long-term dance related training may attenuate age-related decline in proprioceptive bias.

## Introduction

Proprioception can be defined as one’s ability to perceive position sense and motion sense in space (Ribeiro and Olivera, 2007). Accuracy of position sense is most commonly assessed with joint position matching tasks without visual feedback. This is usually tested in two conditions: an *ipsilateral remembered joint position matching task* in which the individual replicates the target joint position; and a *contralateral concurrent joint position matching* *task* whereby the individual matches the target joint position with the other hand (Goble et al., 2009).

Proprioception is essential in sensorimotor control for movement acuity, joint stability, motor coordination, and balance (Mourcou et al., 2015). For example, the position sense of the shoulder joint is important during reaching (Paolucci et al., 2016). Further, improved proprioceptive performance has been linked to improved motor performance (Paolucci et al., 2016). Reaching out to a nearby object without visualizing the object is common during many daily activities (Marini et al., 2016). While proprioception is essential for motor control and joint stability during daily activities and sport activities (Salles et al., 2015), we still have an incomplete understanding of how proprioception differs between joints, and how such differences affect motor control and learning (Marini et al., 2016).

Importantly, balance and proprioceptive function decline with age and these declines are associated with an increase in fall risk in older adults (Sohn and Kim, 2015). In addition, motor coordination also declines with age, which is essential for upper extremity tasks. During an ongoing movement, e.g., reaching for an object, immediate motor corrections occur due to continuous sensory feedback. For successful motor coordination, proprioceptive awareness of the hand and wrist position in space is essential (Cressman et al., 2010; Henriques and Cressman, 2012). In this context, older adults need significantly more time to make corrections when reaching for an object without vision, compared with young adults (Helsen et al., 2016). If distal joint proprioception is impaired, then proprioceptive training might help preserve motor coordination longer in older adults. The direct relationship between age-related decline in upper limb proprioception and upper limb activities in daily life is less known (Adamo et al., 2009; Herter et al., 2014), but given the importance of intact feedback control mechanisms for reaching, grasping and fine motor movements, it is relevant to establish the degree of age-related decline of distal joints, such as wrist proprioception, in older adults.

With respect to distal upper limb joints, limited evidence from small sample studies in older adults (*n* < 30) indicates that there is an age-related decline in active wrist position sense. For example, Boisgontier and Swinnen (2015) reported that proprioceptive bias was larger in older adults in comparison to young adults for joint positions that are far (25°) from, but not for those close to, a neutral wrist position (15°, 5°), while Adamo et al. (2009) found an age-related decline when matching a 40° wrist extension position. Often fine motor tasks require a limited range of motion of the wrist which is why it is important to test proprioceptive acuity over a smaller distance, close to the neutral wrist position. Therefore, the first objective of this study was to confirm earlier findings of an age-related decline of proprioceptive acuity in wrist joints in a larger, more representative sample of older adults. Using established joint position matching paradigms, we assessed the two aspects of joint position accuracy: *proprioceptive bias*, or systematic error, and *proprioceptive precision* (Holst-Wolf et al., 2016), representing a measure of response variability or random error.

Further, there is evidence to suggest that regular physical activity may attenuate the age-related decline in wrist proprioceptive acuity (Adamo et al., 2009; Helsen et al., 2016). Adamo et al. (2009) determined metabolic equivalent values based on seniors’ weekly physical activity levels and demonstrated that wrist joint matching errors were 14% greater in sedentary older adults, compared to their active peers. Other active body-focused training such as Tai Chi also seems beneficial for proprioceptive acuity (Tsang and Hui-Chan, 2003; Tsang et al., 2013; Suetterlin and Sayer, 2014). Therefore we examined which kind of active lifestyle or specific training could help preserve wrist proprioceptive acuity. Thus, the second objective of this study was to determine whether a history of participating in specific types of training (including dance, music or sport) in the past and/or currently participating in regular physical activity (aerobic, strength or balance training) is associated with preserved wrist proprioception. Playing an instrument and performing dance requires significant proprioceptive skills (Jola et al., 2011; Artigues-Cano and Bird, 2014). Dance requires accurate joint position sense and high postural control capacity (Smitt and Bird, 2013). Proprioception is particularly important for ballet and ballroom dance as wrist and hand movements adhere to strict rules of positioning and alignment with the forearm to create a fluid appearance (Daprati et al., 2009).

## Materials and Methods

### Participants

We used convenience sampling to recruit healthy older adults (65 years of age or older) at the Minnesota State Fair and healthy young adults (18–30 years of age) at the University of Minnesota-Twin Cities campus. Older adults voluntarily visited the research boot at the Minnesota State Fair. A sign was available describing the study and the inclusion and exclusion criteria and participants addressed the research staff if they were interested in participating. Fliers were hung around the university campus, describing the study and inclusion/exclusion criteria and young adult students voluntarily contacted the research staff for participation. We accepted every participant who fitted the inclusion and exclusion criteria.

All participants completed a questionnaire on medical history. We asked all participants about sequellae from muscular, cardiopulmonary or neurological diseases or injuries. Participants were included when they were asymptomatic, with no history of any type of injury or shoulder instability, no severe cognitive, severe sensory loss or severe motor impairment. Participants were excluded if they could not move either wrist without pain in full range of motion or feel any wrist movements (assessed with a basic position sense and motion sense test explained below), or if they had cognitive impairments (i.e., <13/16 on the Mini-mental State Exam-2 brief version, MMSE^®^-2-BVTM; Folstein et al., 1975).

The University of Minnesota Institutional Review Board approved the study (IRB#1605S87153). Oral informed consent was obtained in accordance with IRB requirements for studies where names or other identifying information are not recorded. This informed consent was in accordance with the Declaration of Helsinki.

### Physical Activity, Specific Training and Sensorimotor Functional Test Data

The questionnaire, designed for the purpose of this study, contained questions pertaining to the number of years that participants had performed dance, music and/or intensive sport training. The type of dance training was documented. We further asked the participants to elaborate on the number of hours of aerobic exercise, strength training and/or balance training participants performed per week. We divided the seniors into a sedentary and an active group, following the AHA recommended healthy lifestyle guidelines of ±30 min/day of moderate activity (Nelson et al., 2007).

The dominant upper limb was determined with the Edinburg Handedness Inventory (Oldfield, 1971). To capture upper limb motor function, fine motor skills, and dexterity and exclude participants who had problems with actively moving the wrist, arm and hand motor function were assessed with the “Motor Evaluation Scale for Upper Extremity in Stroke Patients,” or MESUPES (Van de Winckel et al., 2006). The MESUPES assesses tone, participation in the movement and active movements of the upper limb and is originally designed to test the affected arm and hand in participants with stroke (Van de Winckel et al., 2006). The MESUPES has established validity (Van de Winckel et al., 2006) as well as repeated established reliability in people with stroke (Van de Winckel et al., 2006; Johansson and Häger, 2012) and can be used for patients with mild to severe arm and hand impairments. Dexterity was assessed with six timed functional tasks per the Jebsen-Taylor Hand Function test, or JTHFT (Jebsen et al., 1969). The JTHFT has been evaluated for reliability and validity (Jette, 1985) and measures fine motor skill and hand dexterity. The six timed functional tasks were simulated page turning, picking up small objects, simulated feeding, stacking checkers, picking up large light objects, and picking up large heavy objects. Normative data, adjusted for age and sex, are based on a very large sample of healthy adults. Somatosensory function was evaluated through tactile touch, position sense, and movement sense of wrist and index finger. Tactile touch is evaluated by assessing tactile sense in the thumb, index and hand. For proprioceptive sensibility, the joint position sense of the metacarpophalangeal joint of the index finger and wrist is evaluated. Three trials were performed per location and per test. Both measures are scored from 0 to 2, reflecting respectively, absent, impaired (one or two mistakes out of three trials), or normal sensory function. Two-point discrimination is tested with the Aesthesiometer^®^ (Lafayette Instrument, Lafayette, IN, USA) and scored for correctly distinguishing one or two pin points in five consecutive trials. The scoring represents the minimal perceived distance in mm, whereby a distance of less or equal than 5 mm reflects intact two-point discrimination; between 6 mm and 10 mm expresses impaired function, and more than 10 mm identifies absent function.

Stereognosis is determined by the number of correctly identified objects a patient can distinguish with their hemiplegic hand and with eyes closed. A total of 6 out of 12 familiar objects are presented to the patient and the number of correctly identified objects is scored, whereby six correctly identified objects represents normal function; four or five correctly identified objects show impaired function; and three or less correctly identified objects reveal absent stereognosis. High inter-rater and test-retest reliability of this clinical evaluation protocol have been established (Klingels et al., 2010).

### Apparatus

**Figure 1:**
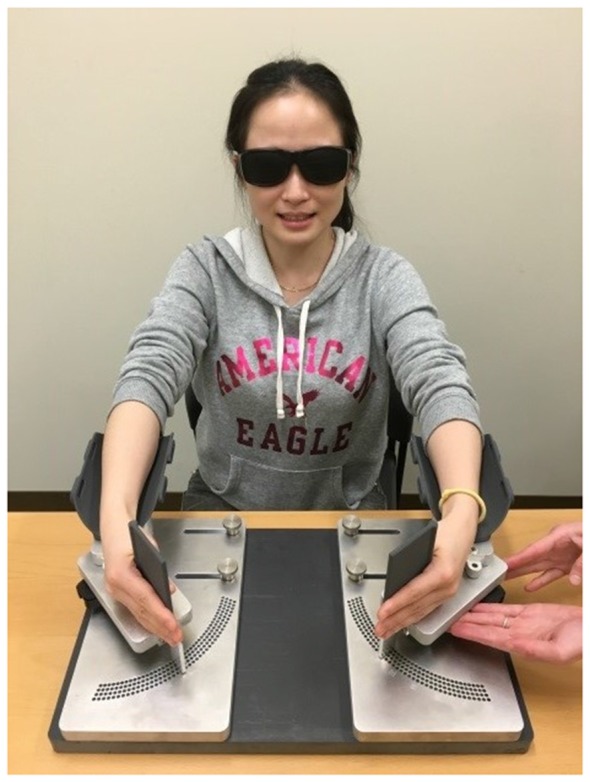
Wrist bimanual manipulandum. Active ipsilateral and contralateral wrist position sense matching of a 15° reference wrist flexion position is performed with occluded vision. Consent was obtained from the individual for the publication of this image.

A wrist bimanual manipulandum allowing wrist flexion/extension movements in the horizontal plane was used for the joint position matching (Figure 1). Two optical shaft encoders (U.S. Digital H6; spatial resolution = 0.036°), housed under the rotating point of the lever arms, recorded the angular position of the hands at a sampling rate of 200 Hz. Participants sat on a height-adjustable chair, and wore glasses that occluded vision. The lever arm length and distance between levers were adjusted to fit the device to the individual anthropometrics of each participant.

### Position Sense Assessment

Two tasks were employed: a contralateral concurrent and an ipsilateral wrist joint position matching task (Adamo et al., 2009). The order of the trials was randomized over the test days. All trials started at 0° wrist flexion with forearms in mid-position between supination and pronation.

During the *contralateral wrist joint position matching*, the experimenter moved the participants’ non-dominant hand in the transverse plane to 15° wrist flexion. The participant then matched the target position with the dominant hand. During the *ipsilateral wrist joint position matching*, the experimenter first moved the participants’ dominant hand to a 15° wrist flexion, held this position for 3 s, and then moved the participants’ hand back to the starting neutral wrist position (0°). The participant reproduced the remembered position with the dominant hand. The dominant hand was used in both conditions based on evidence that the dominant arm makes less execution errors than the non-dominant arm when performing motor tasks (Annett et al., 1979). In both contralateral and ipsilateral matching tasks, researchers maintained the movement speed at 20–25°/s. Movements were recorded from beginning to end. After 2 min of practice, 10 trials were performed (five trials for contralateral and five trials for ipsilateral matching) for a total duration of approximately 10 min. The participants did not receive feedback on their performance.

Wrist proprioceptive acuity was measured through systematic position sense error (PE), or *bias*, while the standard deviation, or random error, indicated the response *precision* (SDP_diff_) across five trials for each task (Holst-Wolf et al., 2016). Absolute bias and signed bias were calculated as the mean angle difference between the active positioned angle by a participant and the 15° reference wrist flexion position. For signed bias, negative values represent undershoot; positive, overshoot.

Different team members were evaluating: (1) current and previous physical activity/training; (2) motor and sensory assessments; and (3) proprioception. The experimenters were blinded to the purpose of the study. The team members testing proprioception did not have information on the physical activity or training levels of the participants. Team members testing the young students did not see the outcome data of the older people and vice versa.

### Data Analysis

The* a priori* sample size calculation was based on *α* = 0.05, Power of 80% and equal proportions of subjects that are in the groups to compare means, with an effect size of 0.56, and standard deviation of the outcome = 1. The result of this calculation was 50 participants in each group^1^. Since we wanted to obtain a large group of older adults to establish the proprioceptive acuity in the wrist, we continued to recruit all available participants.

The normal distribution of the data was assessed with the Shapiro-Wilk test. Both absolute bias and absolute precision for contralateral and unilateral matching were not normally distributed (*p* < 0.0001).

To understand the magnitude of bias in both older and young adult group, we calculated differences in absolute bias and precision with a two-way ANOVA on the ranked data, with group (young, older) and type of task (ipsilateral, contralateral matching) being the independent variables, and degrees of matching error (bias, precision) as the dependent variable. Bonferroni correction was applied for multiple comparisons. Significance level was set at *α* = 0.05.

Relative bias for contralateral matching was normally distributed (*W* = 0.99, *p* < 0.11), but not for unilateral bias (*W* = 0.97, *p* < 0.0018), nor for relative contralateral or unilateral precision. Mixed effect models are more robust for data that do not follow a normalized distribution. Therefore, for signed bias, we applied a longitudinal mixed-effect linear model with sex, handedness, contralateral vs. ipsilateral matching, and age group as fixed effects, and trial number and participant number as random effects. We also examined the effects of sex, handedness and age on proprioceptive relative precision (i.e., variability of five trials with signed bias) with a regression model.

We examined the effects of years of sports, musical, or dance training on proprioceptive signed bias and precision, controlling for trial number, age, sex and contralateral vs. ipsilateral matching using a longitudinal mixed effects model. We performed the same analysis for current exercise training in terms of hours/week of aerobic, balance, or strength exercises. The proportion of people doing various previous and current training was compared between the older and younger group with Fisher Exact tests. We also performed *t*-tests and non-parametric statistics to evaluate the effect of sedentary vs. active lifestyle in older adults on relative and absolute bias and precision.

## Results

We recruited 111 healthy older adults at the Minnesota State Fair. Five people were excluded from the study—two because their MMSE^®^-2-BV^TM^ score was 12/16, two others did not complete the proprioception assessment, and one did not complete the sensorimotor testing. Proprioceptive data was recorded in the remaining 107 older adults (average 70 ± 5 years, range 65–84 years; 57 women).

Most of the older adults were right handed (88%). The majority were white (97%), non-Hispanic (93%) adults. Ninety percent of older adults had received schooling above high school level (bachelor, master level or above; 90%), compared to 10% who only completed high-school. None of them were excluded based on basic testing for motor, sensory or cognitive functions. They scored maximum (95% of the older adults) or almost maximum (5%) on the MESUPES arm-and hand items (86% scored maximum or near maximum). None to very few participants were outside of the normative data for timed dexterity tests on the Jebsen-Taylor test (max 1%–5% was outside of the norm, with exception of 12% of people being slower for scooping beans with a spoon with the dominant hand in males). These high scores on the tests were expected since these tests were originally designed for participants with an affected arm and hand function due to stroke. Twenty-three percent of the older adults had reported a fall at least once in the past year (mostly due to ice and snow in the winter). History of neurological diseases, cardiac and musculoskeletal diseases occurred in 13%, 23% and 48% of the cases. Arthritis (34%) was the most prevalent cause of musculoskeletal disease, but all could move their wrist appropriately, which was relevant for the proprioceptive testing.

Fifty-one healthy young adults (average 20 ± 1 year, range 19–26 years; 27 women) were recruited at the University of Minnesota-Twin Cities campus. Almost all of the young adults were right handed (94%). There was no significant difference in handedness between the older and young adult group (Chi square test, *p* > 0.05). Of all young adults, 84% got a degree in college after high school education. Eighty percent of the young adults were white, and 98% were non-Hispanic. None of the young participants were excluded based on the basic testing for motor, sensory, or cognitive function. Thirty one percent of young adults fell at least once in a year, mostly due to ice and snow in the winter. History of neurological diseases, cardiac and musculoskeletal diseases occurred in 10% (concussion or physical trauma), 2%, and 0% of the cases, with no sequellae. All had a normal wrist range of motion.

After our recruitment was completed, we performed a power calculation with G*Power 3.1.9.2. with the recruited sample sizes of 107 older adults and 51 young adults. For a two-tailed *t*-test with effect size 0.56 and *α* = 0.05, the Power was 0.91.

Physical activity levels were assessed via a questionnaire. Ninety-six percent of young adults participated in aerobic activity, 82% in strength training and 50% in balance training for a minimum of 30 min and maximum of 12 h/week. In the older group, 58% participated in aerobic exercise, 42% in strength training and 21% in balance training for a minimum of 30 min and maximum of 12 h/week. Following the American Heart Association (AHA) guidelines of ±30 min/day of moderate activity (Nelson et al., 2007), we calculated that 88% of young adults were active, and 12% were sedentary compared with 49% of the older adults active and 51% sedentary. The Fisher Exact test revealed that the proportion of young adults who were active was greater than the proportion of older adults who were active (*p* < 0.0001). This significant difference was maintained when analysis was performed for each type of activity (i.e., aerobic, strength and balance training).

Regarding previous training, young adults had some musical (33%), sport (96%) and dance training (22%) ranging between 1–16 years; while for older adults the percentages were 33%, 36%, and 18%, ranging between 3 months and 65 years. According to the Fisher Exact test, there was no significant difference in proportions of musical training (*p* > 0.05) or dance training (*p* > 0.05) between older and young adults, but a greater proportion of young adults had previous sport training (*p* < 0.0001), compared to the older adults. Of all participants, 18% (*n* = 29) of the participants had dance training (19 older adults and 11 young adults). Nine participants in the older adult group had extensive dance training (between 9–65 years). A majority started with ballet in their childhood and transitioned to swing/ballroom-type dancing later in life. About half of the participants with extensive dance training had been professional dancers. Four participants in the young adult group had over 5 years of dance training (between 6 years and 13 years) and four young adults had 4 years of dance training. All did either ballet or swing/ballroom-type dancing.

### Effect of Aging on Proprioceptive Bias

Table 1 displays the Means ± standard deviations and Median (interquartile ranges) of both signed and absolute bias and precision. We calculated the effect sizes (Hodges’ *g*, for two sample *t*-tests with unequal sample sizes) for the relative bias for contralateral and ipsilateral matching in older and young adults, revealing minimal to moderate effect sizes for the difference in means between the two groups (Table 1).

**Table 1:** Absolute and signed values of proprioceptive bias and precision in young and older adults.

	Young adults	Older adults	Effect size (Hedges’*g*)
**Absolute bias** (Median − IQR)			
Contralateral task	3.87 (3.78)	4.80 (5.20)	
Ipsilateral task	2.67 (2.43)	3.59 (2.6)	
**Signed bias** (Mean ± standard deviation)			
Contralateral task	1.84 ± 4.40	4.74 ± 4.69	0.63
Ipsilateral task	2.43 ± 2.96	3.48 ± 2.49	0.40
**Absolute precision** (Median − IQR)			
Contralateral task	2.63 (1.93)	2.06 (1.60)	
Ipsilateral task	1.65 (1.53)	1.89 (1.1)	
**Relative precision** (Median − IQR)			
Contralateral task	3.29 (2.18)	2.34 (1.50)	
Ipsilateral task	2.47 (1.42)^#^	2.14 (1.17)	

*Hedges’ g provides a measure of effect size weighted according to the relative size of each sample. This is used when the groups have different sample sizes. Interpretation: 0.2–0.49: minimal; 0.50–0.79: moderate; 0.80 and above: large effect size. Data that were normally distributed are reported in Mean ± standard deviation; data that were not normally distributed are presented in Median and interquartile range (IQR) = (Q3−Q1). ^#^This distribution in young adults was normally distributed but is reported as Median (IQR) to allow comparison with the older adult group*.

For the ranked data of absolute bias, there was no interaction between type of task and age (*F* = 0.172, *p* = 0.678). The ranked data of absolute bias were not significantly higher in older compared with young adults (*F* = 3.80, *p* = 0.05), but were higher for contralateral vs. ipsilateral matching task (*F* = 15.588, *p* < 0.01).

**Figure 2:**
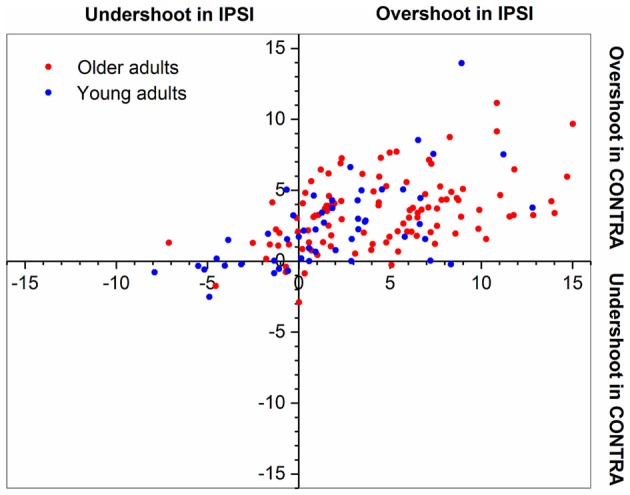
Plot of overshoot/undershoot bias in older and young adults. Proprioceptive bias for both joint position matching tasks. Dots placed at the 0° position would indicate a perfect match. Note that both young and older adults tended to overestimate the 15° reference position. Each data point represents the mean of values for a single participant.

**Figure 3:**
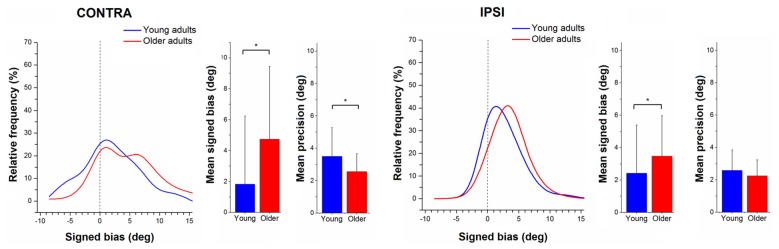
Relative frequency distribution for signed bias. Mean and standard deviation histograms for signed bias and precision during active ipsilateral and contralateral wrist position sense matching. Relative frequency distribution of mean outcomes of proprioceptive signed bias in older (red) and young adults (blue), expressed in percentages. Note that the distribution for the older adults is shifted to the right, indicating a higher bias. Means and standard deviations of contra- and ipsilateral signed bias and precision (in degrees) are shown in the respective bar graphs, with a * indicating a significant difference, *p* < 0.05.

**Figure 4:**
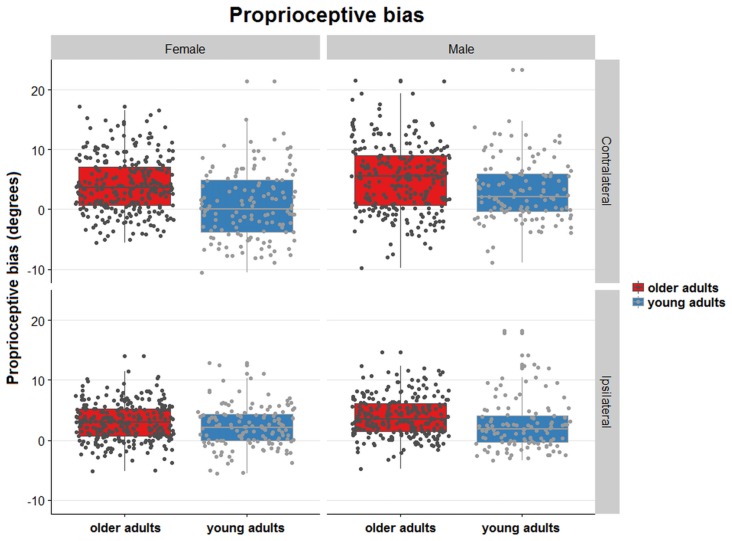
Distribution plots showing proprioceptive signed bias of all trials for contralateral and ipsilateral matching in young and older females and males. Proprioceptive bias for both joint position matching tasks. Each dot represents a single trial. The dots are randomly jittered to avoid point spatial overlap. The degree of error in proprioceptive bias is represented for each type of task, sex, and age group. The colored boxes represent the mean and standard deviation of the proprioceptive signed bias for the type of task sex and age group.

Figure 2 illustrates ipsilateral (X-axis) and contralateral (Y-axis) relative matching errors and their relationship in terms of undershoot and overshoot. Regarding signed bias, both young and older adults tended to overshoot the reference position (66.7% in young adults; 86.9% in older adults) for both types of testing (ipsilateral or contralateral hand; see Figure 2). Bias was lower in young adults compared to older adults (*t* = −4.96, *p* < 0.01, Figure 3). Bias was smaller in the ipsilateral when compared to the contralateral matching task (*t* = −3.83, *p* < 0.01). There were no effects of handedness, sex, or trial number (*p* > 0.05). The difference between the signed and absolute bias value in young adults for contralateral matching is due to young females performing more undershoot errors, compared to other groups, which was evidenced by an interaction between the type of matching task and age (*t* = 5.29, *p* < 0.01, Figure 4).

### Effect of Aging on Proprioceptive Precision

There was no interaction for the ranked data of absolute precision between type of task and age (*F* = 2.014, *p* = 0.16). The ranked data for absolute position sense precision were not significantly different for the older group vs. the younger group (*F* = 1.278, *p* = 0.26), but the error was significantly higher for the contralateral task vs. the ipsilateral task (*F* = 7.91, *p* < 0.005).

**Figure 5:**
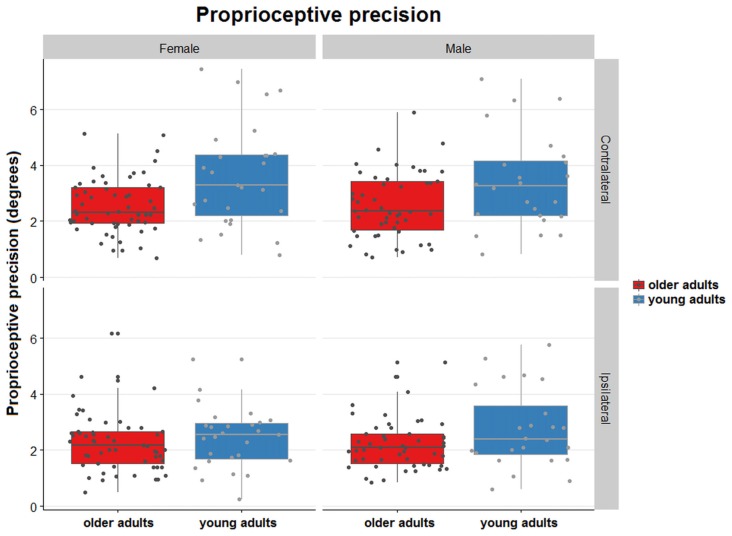
Distribution plots of proprioceptive precision for contralateral and ipsilateral matching in young and older females and males. Proprioceptive precision over the 5 trials/task for both joint position matching tasks. The dots are randomly jittered to avoid point spatial overlap. The degree of error in proprioceptive precision is represented for each type of task, sex, and age group. The colored boxes represent the mean and standard deviation of the proprioceptive precision for the type of task, sex and age group.

For relative precision, the interaction between the type of matching task and age was significant (*t* = −2.73, *p* < 0.01), with the older adults displaying lower precision errors than younger adults. This age-related difference in precision was larger in the contralateral concurrent matching task than in the ipsilateral task (Figure 3). There were no effects of sex or handedness (*p* > 0.05, Figure 5).

### Effects of Physical Activity and Specific Training on Proprioceptive Function in Older Adults

**Figure 6:**
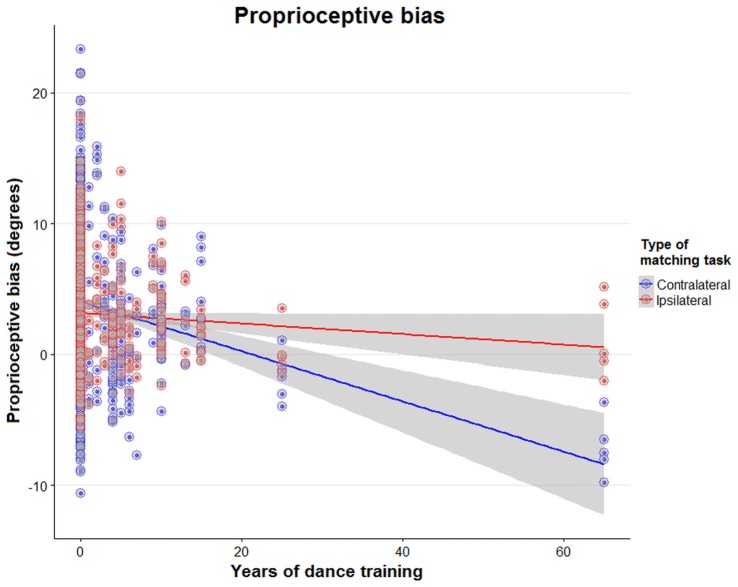
Effect of years of dance training on ipsilateral and contralateral bias. Scatter plot of proprioceptive bias during a contralateral (blue) or ipsilateral (red) matching task in young and old adults, with data plotted against years of dance training. The regression lines show the association between years of dancing and proprioceptive bias in both matching tasks.

Surprisingly, of all training, only longer years of dance training resulted in smaller bias (*t* = −4.85, *p* < 0.01; Figure 6). Examination of the interaction between contralateral vs. ipsilateral matching and dance training (*t* = 5.61, *p* < 0.01) showed that longer dance training resulted in smaller bias (i.e., smaller overshoot errors) in the ipsilateral matching task. Years of sports or musical training did not affect *bias* for either matching task (*p* > 0.05). Similarly, current exercise training in terms of hours/week of aerobic, balance, or strength exercise did not affect proprioceptive bias (*p* > 0.05). Years of sports, musical, or dance training, or hours of current exercise training did not affect *precision* for either matching task (*p* > 0.05).

There was no significant difference for relative bias (*t*-test, *p* > 0.05), relative precision or absolute bias or precision (Mann-Whitney *U* test, *p* > 0.05) when comparing the active vs. sedentary older adult group. We did not perform any analysis on the young adult group because the sample size of sedentary young adults was too small (*n* = 6).

## Discussion

This study reports normative data on contralateral concurrent and ipsilateral wrist position sense in a large group of older functionally independent, community-dwelling adults.

The main findings of this study are: first, aging is associated with a decline in wrist proprioceptive bias and that this decline affects even positions close to the neutral position (15° wrist flexion). Second, aging does not affect proprioceptive precision, meaning that older adults remain consistent in their response behavior despite the fact they tended to exhibit a higher bias. Third, our findings suggest that preservation of wrist proprioception bias may be associated with specific training rather than regular physical activity. We did not find that physical activity, in itself, attenuates age-related decline in proprioception, but we did find potential benefit that prolonged dance training may be associated with a reduced decline in proprioceptive bias.

### The Effect of Aging on Wrist Proprioceptive Function

This study demonstrates that wrist position sense acuity declines with age. Our study aligns with the work by Adamo et al. (2009) and Boisgontier and Swinnen (2015) that proprioceptive bias increases with age. A novel aspect of our study is that we analyzed both aspects of position sense accuracy—bias and precision. Position sense precision, expressing the consistency of positions sense over repeated trials, did not decline in our sample of older adults. That is, aging is associated with a shift in proprioceptive bias, but the response certainty is not affected. The mechanisms contributing to the decline in proprioception in older adults include both peripheral and central changes (Ribeiro and Olivera, 2007). Peripheral changes comprise structural modifications in mechanoreceptors and changes in muscle spindle function. Centrally, ageing has been related to impaired efficiency of feedback processing of sensory information to guide limb target control (Ribeiro and Olivera, 2007; Goble and Anguera, 2010). Age-related physiological and anatomical degenerative effects such as changes in motor unit size, properties, control and morphology as well as altered inputs from the nervous system, can lead to reduced neural control affecting fine motor performance (Erim et al., 1999; Hunter et al., 2016; Sebastjan et al., 2017). With age, motor units reduce in number and become larger in size (Larsson and Ansved, 1995; Ling et al., 2009; Hunter et al., 2016). Further, an increase in variability of discharge rate of motor units at a low force in older vs. young adults, reflects a slower and less steady muscle response (Erim et al., 1999; Hunter et al., 2016). Alterations in the physiological properties of motor units can change the signal-to-noise ratio that muscle afferents receive (Hasson et al., 2016). The decrease in signal-to-noise ratio may originate at the proprioceptor level (Ribeiro and Olivera, 2007; Boisgontier and Swinnen, 2015). Based on this knowledge, we speculate older adults might move slower through these motions allowing them to be consistent in their motion even though they exhibit a higher bias in acuity due to the reduction in signal-to-noise ratio that the muscle afferents receive.

Specifically regarding proprioceptive bias, our data indicate that the tendency to overestimate the true physical limb position increases in older adults. This difference is most pronounced during contralateral matching where bias in older adults is 88% higher when compared with younger adults. Contralateral matching is presumed to be related to interhemispheric transfer of position information (Adamo et al., 2009). In addition, aging is associated with degeneration of the corpus callosum, especially in the rostral part of the genu and isthmus (Ota et al., 2006). Genu and splenium are moderately correlated with upper and lower limb motor tasks (Sullivan et al., 2001) and are highly correlated with bimanual coordination in adults (Johansen-Berg et al., 2007). The correlation between proprioceptive decline and degeneration of posterior midbody, isthmus and splenium is unclear. However, the cortical divisions *corresponding* to these callosal subareas are related to proprioception and shape discrimination tasks (Van de Winckel et al., 2005; Lewis et al., 2009).

### The Effect of Physical Activity and Specific Training on Proprioceptive Function in Older Adults

Our study suggests that preserved wrist proprioception might be associated with dance specific training. As such, it has been shown that 12 weeks of creative dance significantly improved arm positioning, knee position sense, and knee kinesthesia in flexion (Marmeleira et al., 2009). Our findings seem to be in support of others that indicate that dance training may benefit sensory and motor performance. For example, Kattenstroth et al. (2010) demonstrated that a group of 24 older adults with an average of 16.5 years of dance experience showed a better performance on sensory, motor and cognitive performance compared with 38 older adults with no history of dance or sport activities. For example, the older adults with dance training exhibited improved hand and arm movements and steadiness and precision of wrist movements. Although the older adults in our group were active with no apparent or very mild reduced mobility and motor coordination, this finding is encouraging as it may indicate a potential treatment strategy to improve balance, mobility and fine motor coordination.

Of the participants with dance training, all were trained in either ballet or ballroom dancing. The preserved wrist proprioceptive acuity in long-term dancers might be related to the fact that ballet, and also ballroom dancing, are an expressive art form that involves making precise and distinct hand postures (Karin, 2016). Jola et al. (2011) reported that dancers show better integration of proprioceptive signals related to hand positions. Attentive proprioceptive or body-focused training might help maintain the sensitivity of mechanoreceptors and muscle spindles. Hospod et al. (2007) demonstrated a modified sensitivity to movement in muscle spindles during movement trajectory recognition in the absence of visual information. We speculate that in ballet and ballroom dancing, where often arm and hand movements are performed while looking elsewhere, these afferents might become more sensitive, especially to different positions over a small range of motion. Our results further suggest that the proprioceptive acuity preservation in older adults with long-term dance training is apparent for *ipsilateral* wrist position sense. Similarly, young adult expert dancers have superior *ipsilateral* shoulder, elbow and hand position sense, but do not outperform controls in a contralateral position sense task (Ramsay and Riddoch, 2001).

Surprisingly, we did not find an association between wrist proprioception and history of sports or musical training or levels of exercise training. At first glance, this is at variance with the findings of Adamo et al. (2009), who reported that active older adults had better wrist proprioception than sedentary older adults. This might be explained by a difference in parameters that were used for analysis, such that we classified exercise by the type of physical activity and they based their analysis for physical activity on the metabolic equivalent expenditure during exercise. Similar to Adamo et al. (2009), we chose community-dwelling adults and our percentage of sedentary older adults (51%) was similar to that found in other studies involving community-dwelling older adults (Gill et al., 2017). The reason that we did not find preserved wrist proprioceptive acuity with long-term music training might be explained by the fact that musicians use wrist position sense in direct relationship with a force motor task, e.g., pianist pressing the keys or violinists holding the strings or holding the bow. Therefore, even though they become very skilled at visuo-motor tasks, movement speed and movement coordination related to their art (Krampe and Ericsson, 1996), this does not necessarily translate to preserved proprioception. Another key difference between musicians and dancers is that musicians practice their motor skills within a limited number of joints needed for their art and within a limited range of motion. In fact, the limited range of motion and staying in unfavorable physiological postures for a long time during a music performance lead to musculoskeletal health problems and pain (Artigues-Cano and Bird, 2014; and Blanco-Piñeiro et al., 2015). To our knowledge, these side effects related to a limited range of motion or unfavorable posture do not exist in dance. Moreover, increased motor skill and proprioceptive ability in joints needed for musical performance do not generalize to other parts of the body. For example, organists and pianists are not more skilled on position sense of the ankle and postural balance control than people without musical training experience (Rein et al., 2010). Dance on the other hand requires an integration of spatial pattern, rhythm, synchronization to external stimuli and whole-body coordination (Brown et al., 2006). Furthermore dancers must maintain an aligned posture with the wrist in many different body positions, so the improvement in proprioception is seen over the entire body, including lower limb and postural balance. Enhanced proprioceptive feedback strengthens synergistic muscle groups and stabilizes limb coordination, thus contributing to the movement efficiency during dancing (Kiefer et al., 2013). Dancers have better endpoint position matching, upper limb position matching and better integration of local proprioceptive signals compared to non-dancers (Ramsay and Riddoch, 2001; Jola et al., 2011). Dancers, compared to non-dancers, also rely more on proprioceptive information when both visual and proprioceptive information are available. Further, Dieling et al. (2014) reported that dancers keep a good position and motion sense of the upper leg even when fatigued, whereas non-dancers have a worse motion sense when fatigued.

### Limitations

While we chose to investigate a large group of community dwelling adults, we cannot generalize our results to adults who would have more marked motor deficits, reduced mobility or who reside in long-term care facilities. Those adults may benefit the most by proprioceptive preservation with dance training or other body-focused training. Therefore, a future study investigating how specific types of training may influence motor performance in individuals with reduced motor coordination and mobility would be a logical next step. Encouraging evidence from the literature and from a systematic review (Hwang and Braun, 2015), reports that dance, regardless of its style, can significantly improve muscular strength and endurance, balance, and other aspects of functional fitness in older adults. A Cochrane review on improving balance in older people mentioned that some types of exercise (including Tai Chi, Qi Gong, dance, yoga) are moderately effective in improving clinical balance outcomes in older people (Howe et al., 2011). Moreover, Tai Chi was reported to have a beneficial effect on knee and ankle proprioception of older women (Chang et al., 2016).

Second, we recruited participants based on convenience sampling. This sampling method could inherently create a sample bias if only people would choose to participate who feel comfortable and are good at performing proprioceptive tasks. Nevertheless, we still observed a large variation in proprioceptive ability in both groups of participants.

Third, participants were not given feedback on their performances over the five trials. This study did not investigate motor learning. We can therefore only speculate that long-term dance training might help correct position bias because of the frequent knowledge of result through visual and especially through proprioceptive feedback.

Fourth, we presented a cross-sectional study, highlighting the age-dependent differences in wrist proprioception. We can therefore not make strong claims about the mechanisms behind the presented results. Whether long term specific types of training will preserve mechanoreceptor sensitivity with aging would be important to investigate in a longitudinal study. Other factors might also play a role in slowing down age-related degeneration on muscle fibers, motor units and mechanoreceptors, for example genetics, nutrition or other healthy life choices (Liutsko et al., 2014). Longitudinal studies could provide greater inside in the role of long-term (body-focused) exercise, as well as the impact of other factors such as genetics and nutrition.

## Conclusion

This study reports normative data on wrist position bias and precision in a large group of older adults, describing a decline wrist proprioceptive bias but not in proprioceptive precision. We further identified a potential benefit of long-term dance training on preserved wrist position sense bias.

The wrist is involved in many daily activities. Adults with neurological damage such as people with stroke or Parkinson’s disease often have impaired proprioception and motor function, hindering quality of life (Guo et al., 2017). Especially for people with marked proprioception and motor impairment, low-cost body-focused training, such as yoga, Pilates, Tai Chi, or dance might have a beneficial effect on daily life functioning. Preliminary evidence demonstrated that Tai Chi may have a protective effect on age-related decline in bias and movement speed (Tsang and Hui-Chan, 2003; Tsang et al., 2013). Furthermore, in a systematic review, based on the 14 reviewed studies, the authors found a mean improvement rate of 26% on somatosensory, somatosensory-motor, balance and neurophysiological outcome measures when proprioceptive training was conducted in healthy adults (Aman et al., 2015). We encourage further research in this regard. Our findings could be of relevance to a variety of fields, such as physical therapists, dance practitioners and scientists interested in human movement control.

## Author Contributions

AVdW, MLK-R, BO-K and JK significantly contributed to the concept of the work; AVdW, MLK-R and JK oversaw the project; AVdW, MLK-R, Y-TT, DC, KL, ML, ZZ and JB acquired the data; AVdW, JK, TAZ, ZZ, JB, Y-TT and MLK-R were involved in data analysis and interpretation. AVdW wrote the manuscript. All authors provided critical revisions for important intellectual content, approved the final version and agreed to be accountable for all aspects of the work.

## Conflict of Interest Statement

The authors declare that the research was conducted in the absence of any commercial or financial relationships that could be construed as a potential conflict of interest.

## Footnotes

^1^http://www.sample-size.net/sample-size-means/
